# *L. rhamnosus* improves the immune response and tryptophan catabolism in laying hen pullets

**DOI:** 10.1038/s41598-021-98459-x

**Published:** 2021-10-01

**Authors:** Claire Mindus, Nienke van Staaveren, Dietmar Fuchs, Johanna M. Gostner, Joergen B. Kjaer, Wolfgang Kunze, M. Firoz Mian, Anna K. Shoveller, Paul Forsythe, Alexandra Harlander-Matauschek

**Affiliations:** 1 Department of Animal Biosciences, University of Guelph, 50 Stone Road East, Guelph, ON N1G 2W1 Canada; 2 Institute of Biological Chemistry, Biocenter, Center for Chemistry and Biomedicine, Medical University of Innsbruck, Innsbruck, Austria; 3 Institute of Medical Biochemistry, Biocenter, Center for Chemistry and Biomedicine, Medical University of Innsbruck, Innsbruck, Austria; 4 Institute of Animal Welfare and Animal Husbandry, Friedrich-Loeffler-Institut, Celle, Germany; 5 Brain-Body Institute, St. Joseph’s Healthcare, McMaster University, 1280 Main Street West, Hamilton, ON L8S 4K1 Canada; 6 Division of Respirology, Department of Medicine, McMaster University, 50 Charlton Avenue East, Hamilton, ON L8N 4A6 Canada

**Keywords:** Animal behaviour, Animal physiology

## Abstract

In mammals, early-life probiotic supplementation is a promising tool for preventing unfavourable, gut microbiome-related behavioural, immunological, and aromatic amino acid alterations later in life. In laying hens, feather-pecking behaviour is proposed to be a consequence of gut-brain axis dysregulation. *Lactobacillus rhamnosus* decreases stress-induced severe feather pecking in adult hens, but whether its effect in pullets is more robust is unknown. Consequently, we investigated whether early-life, oral supplementation with a single *Lactobacillus rhamnosus* strain can prevent stress-induced feather-pecking behaviour in chickens. To this end, we monitored both the short- and long-term effects of the probiotic supplement on behaviour and related physiological parameters. We hypothesized that *L. rhamnosus* would reduce pecking behaviour by modulating the biological pathways associated with this detrimental behaviour, namely aromatic amino acid turnover linked to neurotransmitter production and stress-related immune responses. We report that stress decreased the proportion of cytotoxic T cells in the tonsils (P = 0.047). Counteracting this T cell depression, birds receiving the *L. rhamnosus* supplementation significantly increased all T lymphocyte subset proportions (P < 0.05). Both phenotypic and genotypic feather peckers had lower plasma tryptophan concentrations compared to their non-pecking counterparts. The probiotic supplement caused a short-term increase in plasma tryptophan (P < 0.001) and the TRP:(PHE + TYR) ratio (P < 0.001). The administration of stressors did not significantly increase feather pecking in pullets, an observation consistent with the age-dependent onset of pecking behaviour. Despite minimal changes to behaviour, our data demonstrate the impact of *L. rhamnosus* supplementation on the immune system and the turnover of the serotonin precursor tryptophan. Our findings indicate that *L. rhamnosus* exerts a transient, beneficial effect on the immune response and tryptophan catabolism in pullets.

## Introduction

In mammals, the rapid colonization of the gastrointestinal tract (GIT) of newborns by microorganisms is heavily impacted by the mother and the surrounding environment^1^. Impairment of this early-life colonization process can disrupt the microbiota composition of the host^2^ and potentially contribute to diseases and psychological disorders later in life^3,4^. Early-life microbial interventions have been used to prevent dysbiosis-related diseases in adulthood^5^. To this end, *Lactobacillus* species are among the most important and widely used probiotics in mammals. While lactic acid bacteria are Gram-positive commensals of the GIT, their abundance is low in humans^6,7^. With numerous reported benefits on the stress response, the immune system, and stress-induced behaviour of mammals, *Lactobacillus*-based treatments are being developed as novel preventives and therapeutics for diseases related to these systems^8–11^.

Similarly, microbial colonization of the chicken GIT plays a key role in immunological and metabolic pathways impacting health and disease^12–14^. The chick gut microbiota undergoes important changes post-hatch^14^. Indeed, the composition and complexity of the gastrointestinal microbial community change rapidly and reach a maximum bacterial density within the first week following hatching^15–17^. In contrast to humans, *Lactobacillus* is a predominant genus throughout the GIT of chickens, and the commensal lactobacilli become established within the first 7 days following hatch^15–18^. They are found in the crop, the proventriculus, the gizzard, the duodenum, the small intestine, and the ceca^14,19–24^. The intestinal *Lactobacillus* population likely originates from the established microbiota of the crop^25^. Lactobacilli resist the low pH and bile salt encountered in the chicken GIT^26^ allowing them to survive the transit through the stomach and duodenum^26^. As such, *Lactobacillus* species are interesting candidates for early-life supplementation aimed at shaping the bacterial community in chickens as: (1) they are a prominent genus within the gut which may translate to a greater role in GIT signaling pathways and (2) they can survive the passage through the GIT if introduced as an oral supplement.

Commensal bacteria exert a range of protective structural and metabolic effects on the GIT^27^. These include nutrient absorption, development of immunity, and competitive exclusion of pathogenic bacteria^18,28^. Indeed, lactobacilli inhibit the growth of pathogens like *Campylobacter*^29^ and improve the numbers of other beneficial microbes^15^. *Lactobacillus* spp. also contribute to chicken intestinal homeostasis by exerting immunomodulatory effects^30–33^. For example, they reshape cytokine expression in chicken cecal tonsils^30–32^ and increase T lymphocyte subpopulations in the GIT of stressed and non-stressed chicks^33^. Furthermore, they modulate the catabolic pathway of the aromatic amino acids (AAA), tryptophan (TRP)^26,34,35^, phenylalanine (PHE), and tyrosine (TYR)^36^. Consequently, lactobacilli influence the level of monoamine neurotransmitters^37^ and the production of neurotransmitters in vivo or in-vitro, such as GABA^38^, serotonin^39^, catecholamines^39,40^, and acetylcholine^41,42^. Alterations to the monoaminergic system by the resident lactobacilli can affect both gut and brain function directly and indirectly.

The metabolic changes associated with inflammatory processes and monoaminergic neurotransmitters play a role in disease^43^ and psychological disorders, such as anxiety or depression^44–47^. *Lactobacillus reuteri* intake improved depressive-like behaviour in mice by reversing the stress-induced decrease of fecal H_2_O_2_ levels and depleting the resident *Lactobacillus* population^35^. The same treatment also increased intestinal IDO-1 expression, the primary enzyme responsible for TRP degradation, and increased plasma kynurenine (KYN) levels^35^, an indicator of TRP degradation away from serotonin production. Furthermore, orally administered *Lactobacillus rhamnosus* reduced stress-induced anxiety- and depression-related behaviour in mice^9,48^. Together, this data suggests that probiotic supplements containing *Lactobacillus* species can improve behaviour, especially under stress^8^.

Interestingly, *Lactobacillus* bacteria have also been linked to severe feather pecking (SFP)^49–51^, a damaging behaviour, often characterized as a psychological disorder in birds^52,53^. While gentle feather pecking (GFP) is a normal part of the laying hen’s natural social behaviour, severe, excessive and repetitive pecking, pulling, and sometimes eating feathers of conspecifics can lead to feather damage, skin injury, and death^54^. SFP etiology is multifaceted with evidence implicating genetic^55–57^ and environmental components^58,59^. It is proposed to be triggered by chronic, unpredictable social or environmental stressors^54^, but the extent and nature of the stress response may differ across genetic lines^60^.

Regardless of the determinants of SFP behaviour, several important observations indicate the involvement of major signaling pathways and the gut microbiota in its pathophysiology in domestic birds. Indeed, birds bred for their high SFP activity are characterized by a lower relative abundance of *Lactobacillus* in the cecal feces compared to non-SFP individuals^50,51^. SFP birds also display distinct AAA metabolism, including higher dopamine and serotonin turnover in the brain, both in the absence of stress^61^ and under acute stress^62^. Other studies show low PHE:TYR and KYN:TRP ratios in stressed SFP birds compared to stressed non-SFP counterparts^63,64^. Furthermore, the gene expression patterns of the neuroendocrine, monoaminergic and immune systems of high peckers are distinct from those of low peckers^60,65–70^.

The *Lactobacillus* genus is significantly underrepresented in the cecal droppings of feather peckers^50,51,71^. Furthermore, *L. rhamnosus* has been demonstrated to attenuate behavioural deficits induced by chronic social stress in mice^72^. Therefore, we originally postulated that oral supplementation with *Lactobacillus rhamnosus* may alter SFP behaviour in chickens. Subsequently, we successfully demonstrated that oral supplementation with *L. rhamnosus* JB-1 prevented SFP in adult chickens^73^. We, therefore, hypothesize that the administration of *L. rhamnosus* to pullets may have a more substantial impact on the microbiota composition. Subsequently, we anticipated changes to stress-induced pecking behaviour, and associated immunological and metabolic changes in laying hens receiving a probiotic supplement. To this end, we investigated whether oral supplementation with the *L. rhamnosus* JB-1 strain during the first nine weeks post-hatch can be a preventive measure against chronic, repeated, and unpredictable stressors later in life. The impact of *Lactobacillus* supplementation was measured by quantifying its ability to prevent SFP behaviour, modulate immunological markers and actors (KYN:TRP ratio, nitrite level, and T cells profile) and affect AAA metabolism (TRP, PHE, TYR, and their respective ratios) in birds selected for and against SFP.

## Results

### Low severe feather-pecking activity in pullets and adult birds

To assess if *Lactobacillus* (Lacto) supplementation can prevent stress-induced severe feather pecking (SFP) behaviour and/or increase gentle feather pecking (GFP), a form of positive social interaction, the incidence of SFP and GFP were recorded for all birds. Pecking behaviour was recorded when birds were pullets (10–13 weeks of age [woa]) and adults (32 woa) to measure both the short- and long-term impact of the supplementation.

The descriptive statistics per treatment groups are listed in Supplementary Table S1, and the odds ratio and statistics are presented in Table 1. The average SFP frequency was 0.051 ± 0.48 pecks/10 min (mean ± SD) in pullets and 0.045 ± 0.34 pecks /10 min in adult birds. On average, 29% of pullets were classified as gentle feather peckers and 16% as severe feather peckers, while 2% of adult birds were classified as gentle feather peckers and 4% as severe feather peckers.

**Table 1:** Odds Ratio (OR) estimates and 95% Confidence Interval (CI), and Chi-square statistics of the short-term (between 10 and 13 weeks of age [woa]) and long-term (32 woa) feather-pecking behaviour in laying hens.

Behaviour	Treatment	Class	Short term (10–13 woa)			Long term (32 woa)	
			OR	95% CI	P-value	Chi-square statistic	P-value
Gentle feather pecking	Supplementation	Placebo	Ref	Ref	0.235	X^2^ _(1, N = 708)_ = 0.529	0.467
		Lacto	0.76	0.483–1.196			
	Stress	S	Ref	Ref	< 0.001	X^2^ _(1, N = 708)_ = 4.551	0.033
		NS	2.57	1.632–4.039			
Severe feather pecking	Supplementation	Placebo	Ref	Ref	0.480	X^2^ _(1, N =708)_ = 3.035	0.082
		Lacto	1.31	0.62–2.765			
	Stress	S	Ref	Ref	0.016	X^2^ _(1, N = 708)_ = 0.542	0.461
		NS	2.52	1.194–5.327			

OR > 1 indicates that birds are more likely to be classified as a feather pecker, whereas an OR < 1 indicates that birds were less likely to be classified as a feather pecker (Feather Pecker: bird that displayed gentle or severe feather pecking at least once between 10–13 woa or 32 woa). Placebo = water supplementation, Lacto = *L. rhamnosus,* S = stressed, NS = non-stressed, Ref = Reference value, n of birds at 10–13 woa: Placebo = 178, Lacto = 176, S = 177, NS = 177 and n of birds at 32 woa: Placebo = 154, Lacto = 157, S = 156, NS = 155.

We report no interaction between supplementation (Lacto, Placebo) and stress (stress, no-stress) treatments on GFP (P = 0.958 at 10–13 woa, P = 0.408 at 32 woa) or SFP (P = 0.424 at 10–13 woa, P = 0.309 at 32 woa). Furthermore, we observed no effect of the supplementation alone in short-term (10–13 woa) or long-term (32 woa) SFP and GFP (Table 1; P > 0.05). The odds ratio (OR) for expressing SFP was significantly higher (OR > 1 indicating increased likelihood of expressing the behaviour) in non-stressed pullets compared to stressed pullets (Table 1). Long-term (32 woa) SFP behaviour remained unaltered by the supplement and stress (Table 1). The odds of expressing GFP were also higher in non-stressed pullets (Table 1) compared to their stressed counterparts. This trend held true in the long-term as well; 87.5% of GFP occurrences in adult birds at 32 woa were observed in the non-stressed group (displayed by only 4% of the birds). The remaining 12.5% of the GFP occurrences were observed in the stressed group and were displayed by 1% of birds (Table 1).

In addition to pecking behaviour, integument damage, an overall indicator of a bird’s body condition, was evaluated by combining the skin injury score and the feather scores into one integument damage index. We report that integument damage was not affected by the supplement type (mean ± SD: Lacto 1.2 ± 1.54 vs Placebo 1.4 ± 1.71), by the presence of stressors (mean ± SD: Non-Stress 1.2 ± 1.53 vs Stress 1.4 ± 1.72) or their interaction (P > 0.05).

Regarding the genetic lines, the OR of expressing GFP (F_2,340_ = 5.43, P = 0.0048) and SFP (F_2,340_ = 4.60, P = 0.011) was significantly different between pullets of the two genetic lines. Birds from the high SFP line (HFP) displayed higher odds for both behaviours than low SFP (LFP) birds (GFP: OR = 2.678, 95%CI = 1.486–4.825, P = 0.003, SFP: OR = 4.344, 95%CI = 1.597–11.820, P = 0.012). At 32 woa, GFP did not differ between lines (X^2^_(2, N =708)_ = 0.270, P = 0.874). The birds from the HFP line performed 71% of the total SFP occurrences observed, while 24% was observed in birds from the unselected control line (UC) and 6% in the LFP line (X^2^_(2, N =708)_ = 12.116, P = 0.002).

### Impact of *Lactobacillus* supplementation on the immune system in response to stress

**Figure 1:**
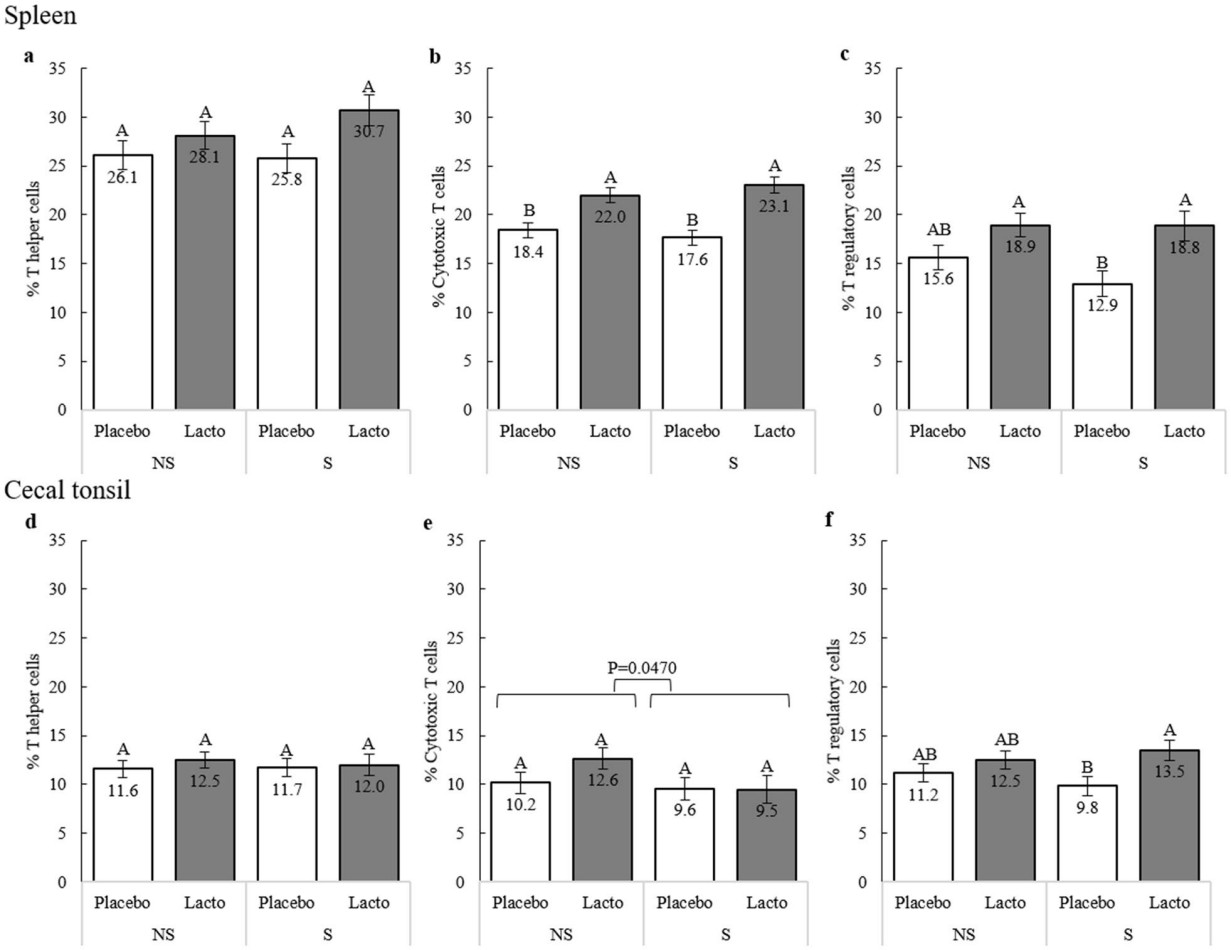
The T cell sub-populations in the spleen and cecal tonsils. Proportions of T cell subpopulations in the spleen (**a**–**c**) and cecal tonsil (**d**–**f**) after nine weeks of supplementation (0–9 weeks of age) and stress treatment (11–13 weeks of age). Sub-populations were identified using the following combinations of cell surface markers: T helper cells = CD3^+^CD4^+^; cytotoxic T cells = CD3^+^CD8^+^; T regulatory cells = CD4^+^CD25^+^ (n of birds: S-Placebo = 9, NS-Placebo = 9, S-Lacto = 9, NS-Lacto = 9, whereby Lacto = *L. rhamnosus,* Placebo = water supplementation, S = stressed and NS = non-stressed) in 15 weeks old birds. Different letters indicate statistically significant differences within the Supplementation*Stress interaction (P < 0.05).

Both stress and psychological disorders, like anxiety and depression, are associated with inflammation^74^. *Lactobacilli* are known to modulate the immune response and to exert anti-inflammatory activity, by modulating T lymphocyte profiles, particularly through enhancement of the regulatory T cell population in the gastrointestinal tract and the spleen, an effect that is more pronounced under stress^33,73,75–77^. The present study assessed the ability of an early-life *L. rhamnosus* supplement to stimulate the avian immune system when challenged by chronic stress. To this end, the proportions of T helper lymphocytes (CD3^+^CD4^+^ T cell), cytotoxic T cells (CD3^+^CD8^+^ T cell), and regulatory T cells (CD3^+^CD4^+^CD25^+^ T cell) in the cecal tonsils and the spleen were analyzed (Fig. 1a–f).

The proportion of splenic cytotoxic T cells was significantly higher in birds receiving the Lacto supplement (Lacto: 22.5 ± 0.56 vs Placebo: 18.0 ± 0.56, F_1,23_ = 31.92, P < 0.0001, Fig. 1b), both in the presence and absence of stress. Lacto treatment also elevated the proportions of splenic T helper cells (Lacto: 29 ± 1.2 vs Placebo: 26 ± 1.1, F_1,23_ = 6.30, P = 0.020, Fig. 1a), and regulatory T cells in both the spleen (Lacto: 19 ± 1.1 vs Placebo: 14 ± 1.0, F_1,23_ = 18.72, P < 0.001, Fig. 1c) and the cecal tonsils (Lacto: 13.0 ± 0.75 vs Placebo: 10.5 ± 0.71, F_1,23_ = 8.22, P = 0.009, Fig. 1f). Nevertheless, the regulatory T cell increase was only statistically significant under stress (Fig. 1 c and f). Animals that experienced stress had a decreased the proportion of cytotoxic T cells in the tonsils (Stress: 9.5 ± 1.13 vs Non-Stress:11.4 ± 0.93, F_1,23_ = 4.41, P = 0.047, Fig. 1e).

### Impact of *Lactobacillus* supplementation on plasma tryptophan

Severe feather-pecking behaviour is modulated by the neurotransmitters serotonin (5-HT) and dopamine^53,61,63,64,78,79^. The concentrations of these neurotransmitters in the blood are influenced by the availability of their aromatic amino acids (AAAs) precursors, tryptophan (TRP), tyrosine (TYR) and phenylalanine (PHE)^80,81^. Here, we investigated the changes to the AAA, KYN and nitrite (immune marker) concentrations in response to Lacto and stress treatment. Changes were monitored at 10, 14 and 32 woa (Table 2).

**Table 2:** Least Squares Means (± Standard Error) of amino acid concentrations, kynurenine and nitrite (n of birds: S-Placebo = 89, NS-Placebo = 89, S-Lacto = 88, NS-Lacto = 88) at 10, 14 and 32 weeks of age (woa).

Supplementation	Lacto		Placebo	
Stress	Stress (n = 88)	Non-stress (n = 88)	Stress (n = 89)	Non-stress (n = 89)
**10 woa**				
Tryptophan (TRP) (µmol/L)	97 ± 1.1 a	97 ± 1.1 a	90 ± 1.1 b	90 ± 1.1 b
Tyrosine (TYR) (µmol/L)	148 ± 2.5	145 ± 2.5	152 ± 2.4	151 ± 2.5
Phenylalanine (PHE) (µmol/L)	95 ± 1.3	94 ± 1.3	92 ± 1.3	98 ± 1.3
TRP:(PHE + TYR)	0.40 ± 0.006 a	0.41 ± 0.006 a	0.37 ± 0.006 b	0.37 ± 0.006 b
KYN:TRP (µmol/mmol)	2.7 ± 0.13	2.9 ± 0.13	2.6 ± 0.13	3.0 ± 0.13
PHE:TYR (µmol/µmol)	0.66 ± 0.011	0.65 ± 0.011	0.62 ± 0.011	0.66 ± 0.011
Kynurenine (KYN) (µmol/L)	0.26 ± 0.012	0.27 ± 0.012	0.23 ± 0.012	0.27 ± 0.012
Nitrite (µmol/L)	31 ± 2.5	36 ± 2.6	28 ± 2.5	34 ± 2.6
**14 woa**				
Tryptophan (TRP) (µmol/L)	99 ± 1.1	98 ± 1.1	98 ± 1.1	99 ± 1.1
Tyrosine (TYR) (µmol/L)	160 ± 2.5	155 ± 2.5	161 ± 2.5	154 ± 2.5
Phenylalanine (PHE) (µmol/L)	100 ± 1.3	98 ± 1.3	100 ± 1.3	98 ± 1.3
TRP:(PHE + TYR)	0.39 ± 0.006	0.39 ± 0.006	0.38 ± 0.006	0.40 ± 0.006
KYN:TRP (µmol/mmol)	3.6 ± 0.13	3.5 ± 0.13	3.5 ± 0.13	3.6 ± 0.13
PHE:TYR (µmol/µmol)	0.63 ± 0.011	0.64 ± 0.011	0.64 ± 0.011	0.65 ± 0.011
Kynurenine (KYN) (µmol/L)	0.35 ± 0.012	0.34 ± 0.012	0.33 ± 0.012	0.35 ± 0.012
Nitrite (µmol/L)	62 ± 2.6	61 ± 2.6	58 ± 2.6	63 ± 2.6
**32 woa**				
Tryptophan (TRP) (µmol/L)	81 ± 1.2	82 ± 1.2	80 ± 1.2	81 ± 1.2
Tyrosine (TYR) (µmol/L)	123 ± 2.6	119 ± 2.6	119 ± 2.6	120 ± 2.7
Phenylalanine (PHE) (µmol/L)	105 ± 1.4	103 ± 1.4	103 ± 1.4	101 ± 1.4
TRP:(PHE + TYR)	0.36 ± 0.006	0.37 ± 0.006	0.37 ± 0.006	0.37 ± 0.006
KYN:TRP (µmol/mmol)	4.0 ± 0.14	3.6 ± 0.14	3.5 ± 0.14	3.6 ± 0.14
PHE:TYR (µmol/µmol)	0.87 ± 0.012	0.88 ± 0.012	0.88 ± 0.012	0.84 ± 0.012
Kynurenine (KYN) (µmol/L)	0.32 ± 0.013	0.29 ± 0.013	0.28 ± 0.013	0.30 ± 0.013
Nitrite (µmol/L)	77 ± 2.7	76 ± 2.7	80 ± 2.7	72 ± 2.7

The birds were submitted to 9 weeks (0–9 woa) of supplementation (Lacto: *L. rhamnosus,* Placebo: water) and 3 weeks (11–13 woa) of stress treatment (stressed, non-stressed). Different letters indicate statistically significant differences within the same row (P < 0.05).

Our results show a significant interaction between supplementation type and time on peripheral plasma TRP concentrations (F_2,471_ = 13.00, P < 0.001, Table 2) and the TRP:(PHE + TYR) ratio (F_2,474_ = 14.96, P < 0.001, Table 2). The latter was used as a proxy for the ratio of total plasma TRP relative to the other large neutral amino acids, an indicator of TRP uptake in the brain and brain 5-HT formation^82^. After 9 weeks of supplementation (10 woa), the Lacto group had higher TRP levels (+ 8%, P < 0.001, Table 2) and a higher TRP:(PHE + TYR) ratio (+ 9%, P < 0.001, Table 2) compared to the Placebo group. Nevertheless, this effect was not long lasting, as there were no significant differences observed between groups at 14 and 32 woa (P > 0.05, Table 2).

We further report that when the data of the three blood collections are pooled (10, 14 & 32 woa), the supplement type and stress treatment interacted to influence the KYN concentration (F_1,469_ = 8.69, P = 0.003) and the KYN:TRP ratio (F_1,471_ = 4.11, P = 0.043). In general, these parameters are more variable in stressed birds. Stressed Lacto birds displayed significantly higher KYN concentrations (Lacto-Stress: 0.315 ± 0.0071 vs Placebo-Stress: 0.283 ± 0.0071, P = 0.007) and a greater KYN:TRP ratio (Lacto-Stress: 3.42 ± 0.080 vs Placebo-Stress: 3.20 ± 0.079, P = 0.177) than the stressed Placebo group suggesting that TRP is preferentially directed to the KYN pathway over the 5-HT pathway in stressed birds receiving the Lacto treatment. Lacto supplementation did not significantly alter KYN or the KYN:TRP ratio in the absence of stress. While the interaction between supplementation and stress did not significantly affect the TRP response, the elevated KYN values in stressed Lacto birds are likely a results of elevated plasma TRP concentrations (P = 0.004). However, it is also possible that the observed result is a result of higher IDO-2 enzyme activity^63^.

### Changes to plasma tryptophan in genotypic and phenotypic feather peckers

In addition to investigating the impact of the Lacto treatment on AAAs metabolism, we explored the association between the genetic lines and AAAs linked to the kynurenine and dopaminergic pathways at 10, 14, and 32 woa (Table 3). We also examined associations between the pecking phenotype (GFP and SFP behaviour) exhibited by the birds between 10 and 13 woa and the aforementioned metabolic pathways (Table 4).

**Table 3:** Least Squares Means (± Standard Error) of aromatic amino acids, kynurenine and nitrite in birds at 10, 14 and 32 weeks of age (woa) (n of birds: UC = 119, LFP = 119, HFP = 116, whereby *LFP* low feather pecking line; *HFP* high feather pecking line; *UC* unselected control line).

	Genetic line		
	UC (n = 119)	LFP (n = 119)	HFP (n = 116)
**10 woa**			
Tryptophan (TRP) (µmol/L)	94.3 ± 0.95 a	98.2 ± 0.95 a	88.2 ± 0.96 b
Tyrosine (TYR) (µmol/L)	156 ± 2.1 a	162 ± 2.1 ab	127 ± 2.1 c
Phenylalanine (PHE) (µmol/L)	97 ± 1.1 a	97 ± 1.2 a	91 ± 1.2 b
TRP:(PHE + TYR)	0.38 ± 0.005 a	0.38 ± 0.005 a	0.41 ± 0.005 bc
KYN:TRP (µmol/mmol)	2.6 ± 0.11 a	2.9 ± 0.11 ab	2.9 ± 0.12 ab
PHE:TYR (µmol/µmol)	0.63 ± 0.009 a	0.60 ± 0.009 a	0.72 ± 0.010 b
Kynurenine (KYN) (µmol/L)	0.25 ± 0.010 a	0.28 ± 0.010 ab	0.25 ± 0.010 a
Nitrite (µmol/L)	34 ± 2.2 a	33 ± 2.2 a	31 ± 2.2 a
**14 woa**			
TRP (µmol/L)	96.9 ± 0.95 a	103.2 ± 0.96 c	96.5 ± 0.98 a
TYR (µmol/L)	162 ± 2.1 ab	170 ± 2.2 b	140 ± 2.2 d
PHE (µmol/L)	100 ± 1.1 ac	100 ± 1.2 ac	96 ± 1.2 ab
TRP:(PHE + TYR)	0.37 ± 0.005 a	0.39 ± 0.005 ab	0.41 ± 0.005 c
KYN:TRP (µmol/mmol)	3.5 ± 0.11 c	3.5 ± 0.12 c	3.5 ± 0.12 c
PHE:TYR (µmol/µmol)	0.63 ± 0.009 a	0.59 ± 0.009 a	0.69 ± 0.010 b
KYN (µmol/L)	0.34 ± 0.010 cd	0.36 ± 0.010 c	0.33 ± 0.011 cd
Nitrite (µmol/L)	61 ± 2.2 b	65 ± 2.2 bc	57 ± 2.3 b
**32 woa**			
TRP (µmol/L)	80.7 ± 1.02 d	81.9 ± 1.04 d	80.4 ± 1.05 d
TYR (µmol/L)	122 ± 2.3 ce	123 ± 2.3 ce	117 ± 2.3 e
PHE (µmol/L)	103 ± 1.2 c	103 ± 1.2 c	103 ± 1.2 c
TRP:(PHE + TYR)	0.36 ± 0.005 a	0.37 ± 0.006 a	0.37 ± 0.006 a
KYN:TRP (µmol/mmol)	3.8 ± 0.12 c	3.8 ± 0.12 c	3.4 ± 0.13 bc
PHE:TYR (µmol/µmol)	0.86 ± 0.010 c	0.85 ± 0.010 c	0.89 ± 0.010 c
KYN (µmol/L)	0.31 ± 0.011 bd	0.32 ± 0.011 bcd	0.27 ± 0.011 ab
Nitrite (µmol/L)	77 ± 2.4 d	76 ± 2.4 d	75 ± 2.4 cd

Different letters indicate statistically significant differences across lines within each time point and across time points within each line for each variable (P < 0.05).

**Table 4:** Least Squares Means (± Standard Error) of the aromatic amino acids, kynurenine, and nitrite in birds at 14 weeks of age according to their feather pecking phenotype (feather pecker: bird that displayed gentle or severe feather pecking at least once between 10–13 weeks of age).

	Gentle feather pecker (n = 103)	Non-gentle feather pecker (n = 251)	P-value
Tryptophan (TRP) (µmol/L)	99 ± 1.2	98 ± 1.0	0.822
Tyrosine (TYR) (µmol/L)	150 ± 2.5	161 ± 1.6	** < 0.001**
Phenylalanine (PHE) (µmol/L)	97.7 ± 0.94	98.9 ± 0.62	0.314
TRP:(PHE + TYR)	0.40 ± 0.006	0.38 ± 0.005	**0.002**
KYN:TRP (µmol/mmol)	3.58 ± 0.108	3.42 ± 0.095	0.549
PHE:TYR (µmol/µmol)	0.664 ± 0.0094	0.627 ± 0.0061	**0.001**
Kynurenine (KYN) (µmol/L)	0.35 ± 0.009	0.36 ± 0.006	0.441
Nitrite (µmol/L)	61 ± 2.3	61 ± 1.5	0.899

	Severe feather pecker (n = 56)	Non-severe feather pecker (n = 298)	P-value
Tryptophan (TRP) (µmol/L)	96.9 ± 1.45	98.8 ± 0.91	0.053
Tyrosine (TYR) (µmol/L)	155 ± 3.5	158 ± 1.5	0.407
Phenylalanine (PHE) (µmol/L)	98.5 ± 1.29	98.5 ± 0.56	0.950
TRP:(PHE + TYR)	0.38 ± 0.007	0.390 ± 0.003	0.367
KYN:TRP (µmol/mmol)	3.7 ± 0.14	3.59 ± 0.086	0.260
PHE:TYR (µmol/µmol)	0.649 ± 0.0130	0.636 ± 0.0057	0.356
Kynurenine (KYN) (µmol/L)	0.35 ± 0.013	0.35 ± 0.006	0.918
Nitrite (µmol/L)	61 ± 3.2	61 ± 1.4	0.966

We found a clear interaction of the genetic line with age in determining the peripheral plasma concentrations of TRP (F_4,471_ = 6.48, P < 0.0001, Table 3), PHE (F_4,473_ = 2.69, P = 0.031, Table 3) and TYR (F_4,469_ = 15.31, P < 0.0001, Table 3), as well as the KYN:TRP ratio (F_4,471_ = 2.66, P = 0.032, Table 3), the PHE:TYR ratio (F_4,465_ = 4.65, P = 0.001, Table 3) and the TRP:(PHE + TYR) ratio (F_4,474_ = 3.45, P = 0.009, Table 3). Overall, HFP birds had higher TRP:(PHE + TYR) and PHE:TYR ratios and lower plasma TRP, PHE and TYR concentrations than birds from the LFP and UC lines (P < 0.05). It is noteworthy that, these differences were only observed at 10 and 14 woa. Overall, KYN concentrations were lower in the HFP birds compared to the LFP birds (F_2,469_ = 6.85, P = 0.001, Table 3).

The AAAs, metabolite and nitrite concentrations based on GFP and SFP phenotype is summarized in Table 4. Gentle feather peckers, who exhibited at least one bout of GFP between 10 to 13 woa, had a higher TRP:(PHE + TYR) ratio (F_1,338_ = 10.11, P = 0.002, Table 4), a lower TYR concentration (F_1,339_ = 15.35, P < 0.001, Table 4) and a higher PHE:TYR ratio (F_1,338_ = 10.82, P = 0.001, Table 4) than birds that did not display this behaviour. On the other hand, birds categorized as severe feather peckers (performing at least one event of SFP between 10 to 13 woa) showed a tendency for lower peripheral plasma TRP (F_1,338_ = 3.76, P = 0.053, Table 4) compared to birds that did not perform this behaviour.

## Discussion

The use of *Lactobacillus* species as a therapeutic for stress-related disorders in humans has gained significant traction over the last decade. In particular, there is an interest in how ingestion of these supplements in childhood or early-life can shape behaviour. The administration of a single *Lactobacillus rhamnosus* strain successfully prevented severe feather pecking (SFP) in adult laying hens^73^, possibly by restoring the lower abundance of *Lactobacillus* reported in SFP birds^50,51,71^. In the present study, we evaluate the ability of the same *L. rhamnosus* strain to act as a preventative measure against SFP when administered in early-life. To this end, pullets received an *L. rhamnosus* supplement for 9 weeks post-hatch prior to undergoing a stress regimen designed to trigger SFP. In doing so, we aimed to elucidate the potential mechanisms of action through which *L. rhamnosus* mitigates stress-induced SFP later in life.

We recorded changes to behaviour and physiological parameters in the short-term (weeks 10–14; i.e., pullets), as well as those that manifest later in life (week 32; i.e., adult birds). The impact of *L. rhamnosus* and stress was measured by quantifying SFP and gentle feather pecking (GFP) behaviour, damage to the feather cover caused by SFP, immunological markers (T cell profiles, KYN:TRP, nitrite) and aromatic amino acid metabolism (tryptophan [TRP], phenylalanine [PHE], tyrosine [TYR], and their ratios). Our data show that early-life *L. rhamnosus* supplementation did not change GFP or SFP frequency and integument damage scores. Overall, *L. rhamnosus* increased the proportions of splenic T helper cells and cytotoxic T cells, as well as splenic and tonsil regulatory T cells under stress, potentially increasing the capacity of the immune response. Notably, the stress regimen decreased cytotoxic T cells within the tonsils. Compared to the placebo, the *L. rhamnosus* supplementation increased peripheral plasma TRP levels and the TRP:(PHE + TYR) ratio in pullets.

Surprisingly, the present study found that the stress treatment did not induce SFP and GFP in pullets. Furthermore, SFP behaviour in pullets and adult birds (Table 1) that received the *L. rhamnosus* (Lacto) supplement was similar to those receiving the placebo treatment (Placebo). It is noteworthy that SFP outbreaks tend to develop in older flocks^83–85^. Moreover, the SFP frequency observed in the present study in both pullets (mean ± SD, 0.051 ± 0.48 pecks/10 min) and adult birds (mean ± SD, 0.045 ± 0.34 pecks/10 min) was 6- to 16-fold lower than that observed in birds of a similar age and from the same genetic lines in previous studies^63,86,87^. For example, pullets of the high SFP (HFP) line and the low SFP (LFP) line performed in average 0.425 and 0.223 pecks per bird per 10 min, respectively^86^. The low SFP incidence was accompanied by no significant changes to the plumage scores, a reliable indicator of SFP behaviour. It is, therefore, likely that a difference between supplementation groups could not be distinguished due to the low incidence of SFP behaviour observed in the current study.

The stress regimen used to induce SFP in this study was designed to mimic the unpredictable and repeated social and environmental stressors that hens encounter in commercial farm settings^88^. Previous research shows that social disruption alone effectively increases SFP in 16- and 24-weeks old laying hens^63,73^. Surprisingly, we report that the odds of expressing SFP during weeks 10–13 were higher in non-stressed birds compared to the stressed birds (Table 1). Nevertheless, it is noteworthy that these higher odds did not lead to changes in integument damage scores. In contrast, no differences in SFP behaviour were discernible in adult hens at week 32 (Table 1). It is possible that, despite the stressors administered, the housing conditions of the birds in the present study were more enriched than the housing environment of birds in commercial systems. Indeed, a lower frequency of SFP is observed in flocks benefiting from enrichment^89^. Between 10 and 13 woa, the stressed birds had limited access to perches and nest boxes, but these items were not entirely restricted. Furthermore, the social disruption stressor may have been perceived as an enrichment instead of a chronic stress, as it allows young birds to interact with new individuals. Indeed, social hierarchies may not yet be fully established, as birds become more territorial, less adaptable, or more easily frustrated as they age^90^. While the stress regimen consisted of a set of social and environmental stressors, the unexpected positive effect of the former may have compensated for the negative stressors. Consequently, it may have contributed to the decreased expression of SFP in stressed birds and the lack of behavioural difference between the stressed and non-stressed groups at 32 woa. From another perspective, if the social mixing represented a positive event at an early age, the birds may have been inadvertently habituated to social changes and less susceptible to its expected negative effect at 32 woa.

Using a model of chronic stress in laying hens^91^ and a supplementation protocol proven effective in rodents^72^, we previously demonstrated that continuous ingestion of *L. rhamnosus* by adult laying hens during a three-week chronic stress treatment reduced SFP^73^. Taken together with the data presented herein, *L. rhamnosus* supplementation buffers the effect of stress on SFP behaviour only when administered concomitantly with the stressors. Similar observations have been made with *L. rhamnosus* JB-1 in a mouse model of chronic social defeat^72^, whereby the probiotic treatment only modulated the behaviour of stressed mice. Therefore, we conclude that a continuous probiotic supplementation would likely be the most effective intervention for stress-induced SFP. Further research is needed to validate if continuous probiotic supplementation in pullets achieves similar results as observed in adult hens.

Despite no significant changes in SFP in response to a preventive probiotic treatment, our data confirm the immunomodulatory effects of *L. rhamnosus.* Birds receiving the probiotic treatment demonstrated increased expression of cytotoxic and helper T cells in the spleen. Additionally, *L. rhamnosus* supplementation led to a larger population of regulatory T (Treg) cells in both the cecal tonsils and the spleen, an effect that was amplified under chronic stress (Fig. 1). Tregs play a fundamental role in the maintenance of immune homeostasis and peripheral tolerance, as well as preventing overwhelming immune responses against invading pathogens. The diversity of the avian gut microbiota affects the complexity of the T cell receptor repertoire in both the gut and the spleen^92^. Oral administration of some microbial organisms is known to modulate immune responses in the lung, the spleen and cecal tonsils^73,76^. *L. reuteri*, now re-identified as *L. rhamnosus* JB-1^93^, induced gut intraepithelial cytotoxic T cells. This effect is proposed to be caused by to the generation of indole derivatives of TRP by the bacterium^94^. Thus, we conclude that early-life supplementation with *L. rhamnosus* enhances the local immune system of hens, similarly to previous observations in mice^77,95,96^ and in adult chickens^73^.

Early-life supplementation of *L. rhamnosus* also increased peripheral TRP concentrations and the TRP:(PHE + TYR) ratio in all birds (Table 2). These results are in accordance with previous data, which suggest that consumption of *Lactobacillus* bacteria enhances amino acid absorption by increasing amino acid transporters in the small intestinal mucosa^97,98^. Notably, the increase in TRP was only observed immediately after supplementation (10 woa). No difference was observed in the short- (14 woa) and long-term (32 woa). This suggests that the impact of *L. rhamnosus* treatment is transient. Given that the optimal effect of *L. rhamnosus* as a probiotic is observed immediately after or during treatment, supplementation appears to be most effective as a curative intervention as opposed to a preventive treatment.

In humans, TRP is mostly catabolized to kynurenine (KYN) by IDO-1, IDO-2 and TDO enzymes. Chickens only possess IDO-2 and TDO but have no gene ortholog for the cytokine-inducible human IDO-1^99,100^. A small portion of TRP is also converted to serotonin (5-HT). In parallel, PHE is catabolized to TYR by the PAH enzyme and then, to L-DOPA, the precursor of catecholamines. In humans, the KYN:TRP and PHE:TYR ratios provide an index of IDO-1 and PAH enzyme activities, respectively^101,102^. Both enzymes are regulated by pro-inflammatory stimuli and stress^46,103^. Interestingly, the higher TRP concentration at 10 woa in birds receiving *L. rhamnosus* was not accompanied by an increased KYN or a higher KYN:TRP ratio. This result contradicts findings in humans, where Alzheimer’s disease patients supplemented with *Lactobacillus*-based probiotics have a significant increase of serum KYN levels, but no change in the concentrations of TRP^104^. Physiological concentrations of TRP and KYN are usually maintained within a relatively narrow range by hepatic tryptophan dioxygenase in mammals^105^. Thus, we hypothesize that the probiotic supplementation increased the TRP concentration, without impacting the KYN pathway. This, in turn, could lead to more TRP being available for 5-HT production, an alternative catabolic pathway^106^ implicated in SFP^53,61,68,70,107^. Additionally, the transient increase of TRP that is preferentially targeted towards the 5-HT pathway may benefit birds’ immune function. In mammals, the IDO-1 enzyme induces an immunosuppressive environment via the degradation of TRP and the accumulation of TRP-derived catabolites (such as KYN), which exert an antiproliferative effect on T cells^108^. However, birds do not possess IDO-1^99^ and likely use the low efficiency isoenzyme IDO-2. This, in turn, may explain the high TRP, but low KYN levels observed in this study. It also accounts for the enhanced proportions of T cells (Fig. 1); however, this theory requires validation in a bird model.

Birds from the HFP line displayed lower peripheral TRP concentrations at 10 and 14 woa compared to birds from LFP and uncontrolled (UC) lines (Table 3). Moreover, birds categorized as severe feather peckers (phenotype) showed a tendency for lower peripheral plasma TRP than birds that did not perform this behaviour. Taken together, both genotypic and phenotypic SFP correlated with low TRP concentrations. In contrast, pullets classified as gentle feather peckers displayed a higher TRP:(PHE + TYR) ratio, a lower TYR level, and a higher PHE:TYR ratio than birds that displayed no GFP. This contradicts Birkl et al.^64^ who found low PHE:TYR ratios in birds displaying aggressive pecking. Another study found that birds with SFP were characterized by higher PHE and a tendency for higher TYR, than the individuals not expressing the behaviour^73^. It is noteworthy that metabolic pathways linked to the "reward" neurotransmitter dopamine are often involved with SFP behaviour^63^ or aggression^64,109^, whereas GFP is often linked to the serotonergic pathways^53,110,111^. This may partially explain why TRP supplementation failed to modulate SFP in some studies^110,112^.

The data presented herein corroborate previous work that implicate the serotonergic system in the development and modulation of GFP and SFP^53,61,68,70,107^. These studies predominantly suggest that low concentrations of brain 5-HT are associated with a predisposition to perform disruptive pecking behaviour^53,113^. While TRP supplementation is reported to reduce GFP, it failed to attenuate SFP^110^. *Lactobacillus* supplementation can be envisaged as a tool to transiently increase TRP concentrations, without the toxicity related to introducing high dietary TRP intake^54,112^. As such, it may be beneficial in birds with a SFP genotype or phenotype.

Finally, some limitations associated with this study must be acknowledged. At 5 woa, the cohort of birds in this study were administered amprolium and penicillin for 6 days to stop the spread of coccidiosis and necrotic enteritis, respectively. The *Lactobacillus* supplementation was not interrupted during the antiparasitic and antibiotic treatments, continuing for three weeks after its cessation. It is noteworthy that antibiotics dramatically alter the gut microbial composition and contribute to intestinal dysbiosis^114,115^. While some species of lactobacilli have a high intrinsic resistance to some antibiotics^116^, their susceptibility to penicillin varies^117,118^. Specific information on the susceptibility of *L*. *rhamnosus* JB-1 to penicillin is currently unavailable. Nevertheless, Simon et al.^115^, showed that dysbiosis induced by a cocktail of antibiotics, including some from the penicillin-type, was remedied two weeks after the end of the treatment. However, we cannot exclude that administering the antibiotic to young birds may have decreased the efficiency of the *L. rhamnosus* probiotic and/or impacted gastrointestinal tract colonization if the strain used in the study was penicillin-sensitive. We further cannot exclude the possibility that *L. rhamnosus* ingestion may have prevented or subdued the dysbiosis in response to antibiotic treatment. Another limitation is that the *L. rhamnosus* supplement was administered in the drinking water to birds as a group, with the exception of hatch day. This methodology was adopted to mimic farming conditions; however, it prevents the calculation of the exact bacterial dose ingested per bird per day. Further work is needed to determine the optimal dosage for *L. rhamnosus* supplementation. Similarly, individual diet consumption was also not measured. Amino acids concentrations are largely controlled by total dietary intake, and thus, variations in feed consumption may have impacted the observed results.

In conclusion, we supplemented pullets with a daily dosage of *L. rhamnosus* to study the impact on the development of SFP in stressed laying hens. Following nine weeks of preventive *L. rhamnosus* supplementation, we observed significant changes to T cell subpopulations and TRP turnover. Nevertheless, the stress treatment did not trigger SFP as intended, which limited our ability to investigate the effects of *L. rhamnosus* supplementation on SFP directly. However, the data suggest that *L. rhamnosus* supplementation is most effective in counteracting SFP behaviour when administered during stress rather than preventatively. As such, the stress-dependent beneficial effects of *L. rhamnosus* is likely due to a buffering effect against stress, via immune system modulation.

## Methods

### Ethical statement

This study was approved by the Animal Care Committee at the University of Guelph (Animal Utilization Protocol #4113). The study was carried out in accordance with relevant guidelines and regulations as well as the ARRIVE guidelines^119^.

### Animals and housing

Eggs used in this study originated from White Leghorn laying hens divergently selected for high (HFP) and low (LFP) severe feather pecking (SFP) activity^57^. A third group of eggs from an unselected control (UC) group of White Leghorns were also used. Eggs were incubated together, and birds were hatched and housed under the same conventional management conditions at the Research Station of the University of Guelph in Guelph, Ontario, Canada. On day of hatch, the birds were sexed and individually wing-tagged. A total of 360 non-beak-trimmed females were systematically allocated to 12 pens of 30 birds each (10 birds of each line). The birds were housed in identical floor pens with wood shavings, one round metal feeder (43 Ø cm), and a drinker line (7 nipples) in a windowless room. From day 1 until 3 weeks of age (woa), a dark brooder (98.5 × 73 × 39 cm) in which 3 sides were enclosed using white “strip door curtains” (allowing movement in and out while providing darkness) was installed in each pen. Each brooder contained a heating pad. At 4 woa, one A-frame perch (15 cm of perch/hen, 55 cm, and 120 cm above the ground) was added to each pen. At 17 woa, three nest boxes were added to each pen. Opaque PVC boards between the pens prevented birds from seeing each other, and only auditory contact was possible. Light was provided at 20 Lux from 05:00 h till 19:00 h and the average daily temperature was 20 °C. Feed and water were provided ad libitum (Research Station feed, Starter: 0 to 6 weeks, Grower: 7 to 16 weeks and Layer: from 17 weeks). The composition and nutrients formulation of the feed are detailed in Supplementary Tables S2 and S3.

At 5 woa, coccidiosis (*Eimeria*, protozoa) and necrotic enteritis (*Clostridium perfringens*) caused the death of 4 chicks. The remaining birds were immediately treated with 0.024% amprolium (Amprol ®, Huvepharma Canada Corporation Inc., Canada) and 0.4 g/L penicillin G potassium (Pot-Pen ®, Vetoquinol N.-A. Inc., Canada) administered via drinking water for 6 days. At 11 woa, two birds were removed from the experiment due to sexing errors. Between 12 to 14 woa, four birds had to be euthanized for health reasons. At 15 woa, one other bird was euthanized because of cannibalistic pecking injuries.

### *Lactobacillus rhamnosus* supplementation and chronic, unpredictable stress treatments

**Figure 2:**
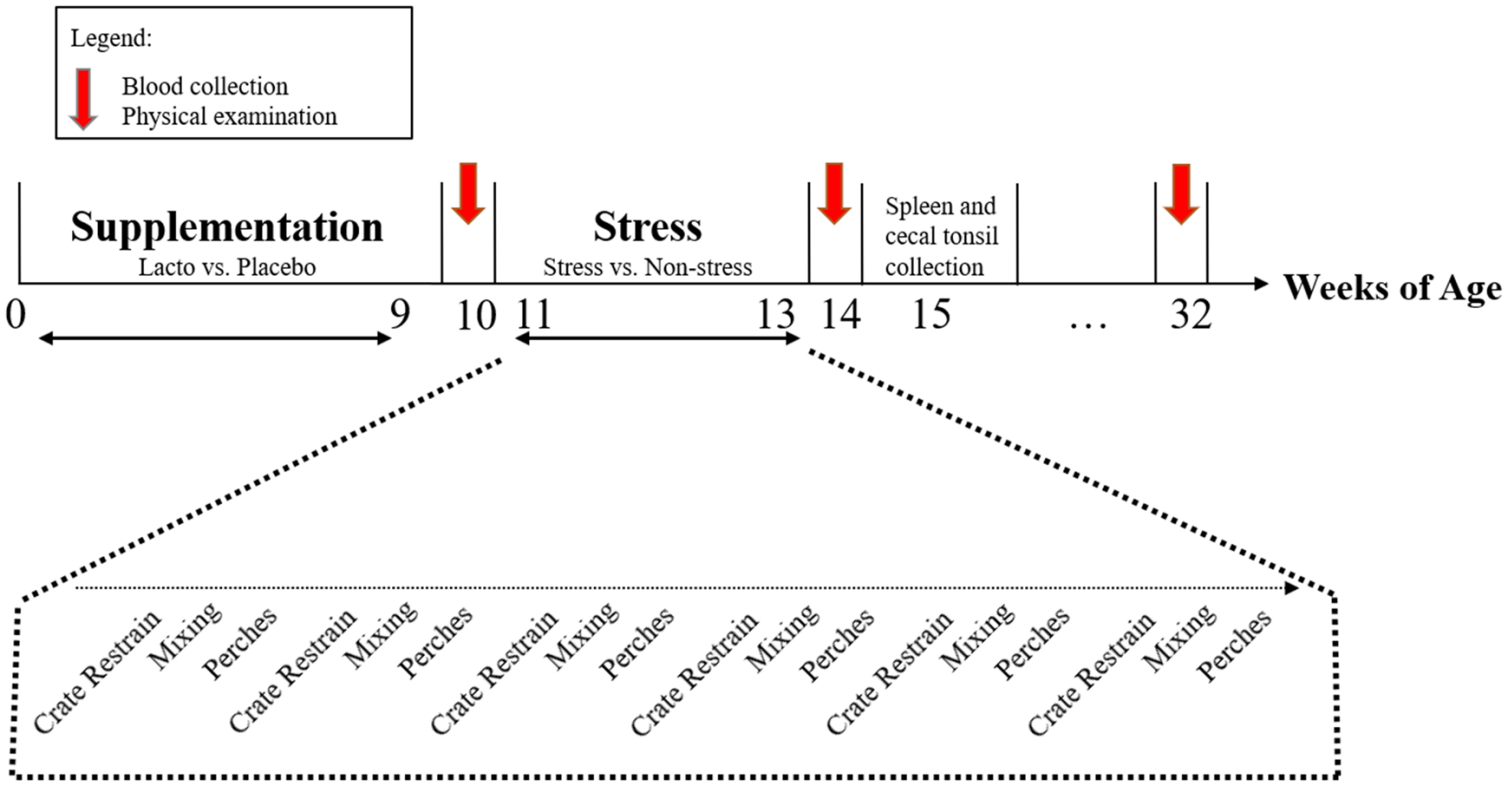
Schematic diagram of the experimental timeline. The Lacto or Placebo supplementation started when birds were 1 day old and lasted 9 weeks. The stress treatment spanned weeks 11–13. Physical examinations and measurements of behaviour, blood samples, and cecal droppings samples were conducted at 10, 14, and 32 weeks of age. Spleen and cecal tonsil samplings were performed at 15 woa from 36 animals.

An overview of the experimental timeline is presented in Fig. 2. Half of the pens were systematically assigned to receive an oral supplement of *Lactobacillus rhamnosus* JB-1™ (Lacto, n = 6 pens, 176 birds) dissolved in water. The other half received a placebo of drinking water (Placebo, n = 6 pens, 178 birds). *L. rhamnosus* JB-1 was a gift from Alimentary Health Inc., Cork, Ireland to Paul Forsythe and Wolfgang Kunze, McMaster University. To inoculate the bird with a sufficient amount of bacteria once to facilitate colonialization of the gut, on day of hatch, chicks of the Lacto treatment group were individually supplemented with 5 × 10^9^ CFU of *L. rhamnosus* JB-1™ dissolved in 0.5 mL of water, while the Placebo chicks received 0.5 mL of water, using a 5 mL plastic syringe. During the following 9 weeks, birds were supplemented as a group at the pen level (Monday to Friday, between 9:00 h and 10:30 h). A dose of 5 × 10^9^ CFU of *L. rhamnosus* JB-1 per bird was dissolved into drinking water. One hour prior to supplementation, the drinker lines were raised to prevent access, and two round 1 L drinkers containing either the Lacto or Placebo treatments were placed in the pens. The birds were allowed to voluntarily consume the respective supplement. Individual consumption was not measured. After consumption of the supplements (in approximately 10-15 min), the round drinkers were removed from the pens and the regular drinker lines were lowered immediately to allow routine access again.

At 11 woa, a stress regimen was administered to trigger SFP^63^. Three pens of each supplementation type were randomly assigned to undergo a sequence of stressors spanning a period of 3 weeks (S, n = 6 pens, 177 birds) while the other three pens were left undisturbed (NS, n = 6 pens, 177 birds) (Fig. 2). Stressors were environmental (physical restraint of all individuals in a pen for 1 h in a transport crate and removal of roosting perches for 24 h) and social (social disruption by mixing). The stressors used in the present study are reported as potential SFP triggers by previous work^63,88,120^. During social disruption, birds from one pen were divided into two subgroups of 14 to 15 individuals and mixed with another subgroup from a different pen receiving the stress treatment and the same supplement (Lacto or Placebo). Upon mixing, birds were placed in a new but identical pen with fresh shavings to create a new environment for all birds. Each stressor was applied in the afternoon and repeated 5 times. The type of stressor used was balanced over the 3-week period, whereby one stressor was administrated each day between Monday-Friday (Fig. 2). This stress regimen was designed to mimic the unpredictable and repeated stressors that hens encounter in commercial farm settings.

### Behavioural observations and physical examinations

Behavioural observations were conducted via video recordings. A camera (Samsung SNO-5080R, IR, Samsung Techwin CO., Gyeongi-do Korea) with an aerial view was installed in each pen. All birds were individually identified using continuously numbered silicone backpacks (8 × 6 × 0.5 cm), fastened around the wings via two elastic straps secured to the backpacks with metal eyelets (Harlander-Matauschek et al., 2009). The time windows used to observe SFP activity were determined by pilot observations. To measure the short-term impact of the supplementation, pens were video recorded for a total of 80 min during the 3-weeks stress treatment: 10 min in the morning for three days spread over the three weeks and 10 min immediately after each mixing stressor. To assess the long-term impact of the supplementation, two more 10-min recordings were made in the morning at 32 woa. Recordings were scheduled outside of the working hours of the farm staff. Behavioural analysis was performed by six blinded observers, trained beforehand (total of 1.7 h of observations for each of the 354 birds and total of 20 h of video). Pearson’s correlations were calculated for intra (r = 0.8754) and inter (r = 0.747) observer reliability and were measured through the video observations.

All-occurrence sampling was used to record the actor and the recipient of gentle and severe feather pecking. A sequence of gentle feather pecks (> 4 s) at the tips and edges of feathers of another bird was considered as gentle feather pecking (GFP)^64^, while intent, forceful peck(s) towards the feathers/body of another bird that may remove feathers was considered as severe feather pecking (SFP). Birds exhibiting at least one bout of GFP or one severe feather peck were categorized as gentle, or severe peckers, respectively, for the short- (10–13 woa) and long-term (32 woa). Non-severe peckers and non-gentle peckers were defined as birds that performed 0 bouts or pecks of the respective behaviour.

A physical examination of each individual bird was completed during weeks 10, 14, and 32 (Fig. 2) to determine feather cover, and injuries. The severity of damage to the feather cover on the neck, back, and tail was assessed on a 0 to 3 scale (0: no or slight wear, nearly intact feathering; 1: damaged feathers or at least one featherless area < $2 Canadian coin [diameter of 28 mm], 2: at least one featherless area ≥ $2 Canadian coin and, 3: at least one featherless area ≥ $2 Canadian coin with fresh bloodstains) adapted from Decina et al.^121^. Pecking injuries on the comb/head were recorded as present or absent.

### Blood sampling and amino acid analysis

At 10, 14, and 32 woa, one-hour post-feeding, blood samples (2 ml/hen) were collected (between 10:00 h and 14:00 h) from the wing vein of each hen into 2 mL EDTA tubes. The tubes were gently inverted to ensure complete mixing before being stored on ice until the end of sampling (maximum 4 h). At the end of sampling, samples were transported (10 min) to the Department of Animal biosciences of the University of Guelph where the plasma was separated by centrifugation at 4 °C, 2,500 rpm for 15 min, and stored at -80 °C until further analysis.

The concentrations of amino acids and their derivatives, and nitrite were determined as reported previously as per Mindus et al.^73^. In brief, samples were analysed via reversed-phase HPLC. TRP, PHE and TYR concentrations were determined by monitoring their natural fluorescence at an excitation wavelength of 286 nm (TRP) and 210 nm (PHE, TYR), and an emission wavelength of 366 nm (TRP) and 302 nm (PHE, TYR). KYN was detected at a wavelength of 360 nm.

In mammals, the KYN:TRP ratio can be used to estimate TRP metabolism along the KYN axis. In humans, this ratio is used as an index of the IDO-1 enzyme-mediated TRP breakdown when accompanied by an increase in markers (such as neopterin) of the cellular immune system^101^. PHE:TYR ratios indicate phenylalanine 4-hydroxylase (PAH) activity, which converts PHE to TYR^102^. TRP:(PHE + TYR) is a substitution for the commonly used ratio of TRP to the large neutral amino acids. As described in Wurtman et al.^81^, this ratio indicates the competition of TRP with other amino acids for uptake across the blood–brain-barrier. As a surrogate marker for nitric oxide (NO) production, the stable NO metabolite nitrite was measured in the plasma sample collected using a modified Griess assay (Merck KGaA, Darmstadt, Germany).

### Flow cytometry analysis for T-lymphocyte profiles

At 16 woa, 36 hens (3 hens per line x supplementation type x stress treatment groups) were killed. Within 3 min after death, cecal tonsil and spleen tissues were collected from each bird in 5 mL of RPMI medium containing 5% fetal bovine serum (FBS) in 15 mL falcon tubes and kept on ice. Cells from both tissues were isolated, suspended, centrifuged, and counted as per Mindus et al.,^73^. Viable spleen and cecal tonsils cells were diluted in Fluorescence-Activated Cell sorting (FACS) buffer (PBS + 2% FBS) to a concentration of 10^6^ cells/ml. Both splenocytes and cecal tonsil cells were stained for T-helper cell (CD3^+^CD4^+^ T cell), cytotoxic T lymphocyte (CD3^+^CD8^+^ T cell) and regulatory T cell (CD4^+^CD25^+^ T cells) markers using the same antibodies as in Mindus et al.^73^. Data were acquired using FACSCelesta (Becton Dickinson, Oakville, ON, Canada) and analysed by FlowJo (BD Bioscience, Ashland, OR, USA). The gating strategy followed the protocol used in Mindus et al.^73^.

### Statistical analysis

Frequencies of behaviours were determined per individual per 10 min. Due to the low frequency of pecking, the behavioural data was analyzed using a binary scale depending on whether or not birds had performed the behaviour in the short- (10–13 woa) or long-term (32 woa) within the course of the experiment. Similarly, the neck, back and tail feather cover scores and the comb injury scores were combined into a new index titled integument damage which reflected presence (at least one score > 0) or absence (all regions scored 0) of damage. To further identify the physiological pathways linked to the behaviour, we categorized birds as gentle feather peckers or severe feather peckers based on whether or not they performed the behaviour between weeks 10 to 13.

Generalized linear mixed models were used to analyze the data collected during this experiment. Variance of GFP and SFP on the short term (weeks 10–13) was partitioned into the fixed effect of Supplementation (Lacto, Placebo), Stress (Stress, Non-Stress), Line (HFP, LFP, UC), and their interaction with a binary distribution. The interaction between the number of birds in a pen at each observation time point and the day of observation was designated as a random effect in the behaviour analysis, with the birds being an experimental unit. Because of zero-biased data, a chi-square test of independence was performed to examine the relation between GFP and SFP displayed over the long term (week 32) and the supplementation, stress, and line. Similarly, variance of the integument damage was partitioned into the fixed effect of supplementation, stress, and line, week of collection (10, 14 and 32) and their interaction with a binary distribution.

Variance of each T-cell subset was partitioned into the fixed effect of supplementation, stress, line, and their interaction using a normal distribution. Pen of the bird was included as a random effect.

Variance of each aromatic amino acid (AAA), their metabolites, and ratios was partitioned into the fixed effect of supplementation, stress, line, week of collection (10, 14 and 32) and their interaction with a normal distribution. In the model analyzing the AAA, metabolites and ratios, the pens of the birds were designated as a random effect with the birds classified as an experimental unit and the three collection times were considered as repeated measures. To identify whether AAA were interrelated with pecking behaviour, an additional generalized linear mixed model was performed for each AAA, their metabolites and ratios obtained at 14 woa with the behavioural phenotype displayed from week 10 to 13 as a fixed effect and the pen as a random effect.

All statistical analyses were performed in SAS (Windows version 9.4, SAS Institute, Cary NC). Least squares means and standard errors on the data scale were obtained using the ILINK option. Differences between means were compared pairwise using a Tukey–Kramer adjustment. Scatter plots of studentized residuals against predicted values and treatment values and a Shapiro–Wilk test of normality were used to confirm the assumptions of the variance analysis. To detect possible outliers, studentized residuals outside a ± 3.4 envelope were used. A type I error rate of 0.05 was applied for all statistical tests.

## Supplementary Information

Supplementary Information.

## Data Availability

The datasets generated during and/or analysed during the current study are available from the corresponding author on reasonable request.
