# Supplementary material for: L. rhamnosus improves the immune response and tryptophan catabolism in laying hen pullets
Source: Sci Rep. 2021 Oct 1;11:19538. doi: 10.1038/s41598-021-98459-x (PMC8486881; doi:10.1038/s41598-021-98459-x)
Supplement: Supplementary file 1 — Supplementary Information. [file 41598_2021_98459_MOESM1_ESM.docx]

***L. rhamnosus* improves the immune response and tryptophan catabolism in laying hen pullets**

Claire Mindus, Nienke van Staaveren, Dietmar Fuchs, Johanna M. Gostner, Joergen B. Kjaer, Wolfgang Kunze, M. Firoz Mian, Anna K. Shoveller, Paul Forsythe and Alexandra Harlander-Matauschek

Supplementary Materials:

Supplementary Tables:

**Supplementary Table S1.** Descriptive statistics of the short-term (between 10-13 weeks of age [woa]) and long-term (32 woa) feather pecking behaviour in laying hens. The pecking frequency per bird per 10 min and the standard deviation (SD) and the percentage of feather peckers in each group are listed (Feather Pecker: bird that displayed gentle or severe feather pecking at least once between 10-13 woa or 32 woa). Observations were recorded following early-life supplementation (weeks 0-9). Placebo = water supplementation, Lacto = *L. rhamnosus,* S = stressed, NS = non-stressed, n of birds at 10-13 woa: S-Placebo = 89, NS-Placebo = 89, S-Lacto = 88, NS-Lacto = 88 and n of birds at 32 woa: S-Placebo = 77, NS-Placebo = 77, S-Lacto = 79, NS-Lacto = 78.

|  |  |  | **Short Term (10-13 woa)** | | **Long Term (32 woa)** | |
| --- | --- | --- | --- | --- | --- | --- |
| **Behaviour** | **Treatment** | **Class** | **Means ± SD** | **Feather Peckers** | **Means ± SD** | **Feather Peckers** |
|  |  |  |  |  |  |  |
| **Gentle Feather Pecking** | Supplementation | Placebo | 0.065 ± 0.319 | 34% | 0.008 ± 0.0915 | 2% |
|  |  | Lacto | 0.058 ± 0.335 | 25% | 0.017 ± 0.1500 | 3% |
|  |  |  |  |  |  |  |
|  | Stress | S | 0.036 ± 0.262 | 18% | 0.006 ± 0.1063 | 1% |
|  |  | NS | 0.087 ± 0.380 | 41% | 0.020 ± 0.1394 | 4% |
|  |  |  |  |  |  |  |
|  | Supplementation * Stress | Lacto-S | 0.031 ± 0.275 | 15% | 0.011 ± 0.1508 | 1% |
|  |  | Placebo-S | 0.041 ± 0.248 | 21% | 0.0 ± 0.0 | 0% |
|  |  | Lacto-NS | 0.084 ± 0.385 | 35% | 0.023 ± 0.1495 | 5% |
|  |  | Placebo-NS | 0.090 ± 0.375 | 46% | 0.017 ± 0.1291 | 3% |
|  |  |  |  |  |  |  |
| **Severe Feather Pecking** | Supplementation | Placebo | 0.045 ± 0.538 | 13% | 0.028 ± 0.2794 | 2% |
|  |  | Lacto | 0.057 ± 0.431 | 19% | 0.063 ± 0.3872 | 6% |
|  |  |  |  |  |  |  |
|  | Stress | S | 0.028 ± 0.262 | 11% | 0.037 ± 0.3035 | 3% |
|  |  | NS | 0.073 ± 0.637 | 20% | 0.054 ± 0.3687 | 5% |
|  |  |  |  |  |  |  |
|  | Supplementation  *  Stress | Lacto-S | 0.033 ± 0.312 | 11% | 0.028 ± 0.1980 | 5% |
|  |  | Placebo-S | 0.024 ± 0.201 | 11% | 0.045 ± 0.3806 | 2% |
|  |  | Lacto-NS | 0.081 ± 0.523 | 26% | 0.097 ± 0.5091 | 7% |
|  |  | Placebo-NS | 0.066 ± 0.733 | 15% | 0.011 ± 0.1057 | 2% |

**Supplementary Table S2.** Ingredient composition of starter, grower and layer phase poultry feed for White leghorn laying hens housed in floor pens. Amounts are expressed in kg/ton feed unless otherwise specified.

| **Ingredient Name** | **Starter 0-6 weeks** | **Grower 7-16 weeks** | **Layer from 17 weeks** |
| --- | --- | --- | --- |
| Corn-Chop | 309.05 | 418.30 | 555.00 |
| Wheat-Chop | 150.00 | 150.00 | . |
| Soybean Meal | 130.00 | 41.00 | 166.00 |
| Wheat Shorts | 95.00 | 150.00 | 51.00 |
| Bakery Meal | 83.00 | . | . |
| Pork Meal | 80.00 | 80.00 | 70.00 |
| Dried Distill Gr & Sol | 75.00 | 75.00 | . |
| Canola Meal | 50.00 | 65.00 | . |
| Calcium Carbonate (Limes) | 9.50 | 9.50 | 95.00 |
| Hi Pro Corn Gluten Meal | . | . | 25.00 |
| Tallow (Av Blend) (Mixer) | 5.00 | . | 10.00 |
| Tallow (Av Blend) (Pelleter) | . | . | 15.00 |
| Monocalcium Phosphate | 4.00 | 2.50 | 5.50 |
| Alimet Liquid (88%) | 1.80 | 1.10 | 1.00 |
| L-Lysine 50% (Liquid) | 1.40 | 2.40 | . |
| Vitamin E (50 kIU/kg) | 1.20 | . | . |
| Fine Salt | 1.10 | 2.20 | 3.00 |
| Rac Broiler Micro #203 | 1.00 | 1.00 | 1.50 |
| Rac Vitamin Booster | 1.00 | 0.50 | 1.50 |
| Availa-4 | 0.75 | 0.50 | . |
| Superzyme Cs Enz | 0.50 | . | . |
| Hy.D Premix | 0.30 | . | . |
| Choline Chloride 70% Liquid | 0.30 | 0.30 | 0.50 |
| Sodium Bicarbonate | . | 0.30 | . |
| Threonine | 0.10 | 0.20 | . |
| Hy.D Premix | . | 0.20 | . |

**Supplementary Table S3.** Nutrient formulation of starter, grower and layer phase poultry feed for White leghorn laying hens housed in floor pens.

| **Nutrient Name (Units)** | **Starter 0-6 weeks** | **Grower 7-16 weeks** | **Layer from 17 weeks** |
| --- | --- | --- | --- |
| Weight (kg) | 100.00 | 100.00 | 100.00 |
| M.E. Poultry (kcal/kg) | 2979.54 | 2877.85 | 2886.30 |
| Crude Protein (%) | 20.92 | 18.01 | 18.07 |
| Arginine (%) | 1.24 | 1.02 | 1.12 |
| Lysine (%) | 1.05 | 0.90 | 0.89 |
| Methionine (%) | 0.47 | 0.38 | 0.38 |
| Tsaa (%) | 0.81 | 0.68 | 0.64 |
| Tryptophan (%) | 0.25 | 0.20 | 0.19 |
| Threonine (%) | 0.72 | 0.62 | 0.63 |
| Glycine (%) | . | . | 1.01 |
| Histidine (%) | . | . | 0.46 |
| Leucine (%) | . | . | 1.60 |
| Isoleucine (%) | 0.74 | 0.60 | 0.70 |
| Phenylalanine (%) | . | . | 0.86 |
| Phenyl + Tyro (%) | . | . | 1.47 |
| Valine (%) | 0.94 | 0.81 | 0.86 |
| Glyc. + Serine (%) | . | . | 1.72 |
| Proline (%) | . | . | 1.14 |
| Crude Fat (%) | 4.74 | 4.32 | 5.77 |
| Linoleic Acid (%) | 1.63 | 1.67 | 1.97 |
| Calcium (Phytase) (%) | 1.08 | 1.03 | . |
| Crude Fibre (%) | 3.08 | 3.43 | 1.83 |
| Adf (%) | . | . | 2.91 |
| Ndf (%) | . | . | 7.63 |
| Dry Matter (%) | . | . | 89.38 |
| Calcium Total (%) | 1.06 | 1.01 | 4.22 |
| Phos. Total (%) | 0.77 | 0.76 | 0.65 |
| Phos Av. Poultry (%) | 0.47 | 0.45 | 0.44 |
| Sodium (%) | 0.18 | 0.17 | 0.18 |
| Chloride (%) | 0.24 | 0.25 | 0.27 |
| Potassium (%) | . | . | 0.61 |
| Magnesium (%) | . | . | 0.17 |
| Sulphur (%) | . | . | 0.22 |
| Iron (mg/kg) | . | . | 209.50 |
| Manganese (mg/kg) | . | . | 119.25 |
| Zinc (mg/kg) | . | . | 118.36 |
| Copper (mg/kg) | . | . | 12.71 |
| Iodine (mg/kg) | . | . | 1.50 |
| Selenium (mg/kg) | 0.30 | 0.30 | 0.45 |
| Cobalt (mg/kg) | . | . | 0.03 |
| Fluorine (mg/kg) | . | . | 9.90 |
| Vit. A (kIU/kg) | 10.00 | 9.00 | 15.00 |
| Vit. D3 (kIU/kg) | 4.65 | 4.10 | 4.50 |
| Vit. E (IU/kg) | 100.00 | 30.00 | 60.00 |
| Menadione (Vit. K) (mg/kg) | . | . | 3.75 |
| Vit. B12 (mcg/kg) | . | . | 37.50 |
| Riboflavin (mg/kg) | . | . | 14.25 |
| Niacin (mg/kg) | . | . | 75.00 |
| Choline (g/kg) | 1.54 | 1.48 | 1.11 |
| D-Pantothenic Acid (mg/kg) | . | . | 24.00 |
| Pyridoxine (mg/kg) | . | . | 6.75 |
| Thiamine (mg/kg) | . | . | 3.75 |
| Folic Acid (mg/kg) | . | . | 3.75 |
| Biotin (mcg/kg) | . | . | 225.00 |
| Deleted (%) | . | . | 0.37 |
| Deleted (%) | . | . | 0.78 |
| Deleted (%) | . | . | 0.63 |
| Deleted (%) | . | . | 0.16 |
| Deleted (%) | . | . | 0.59 |
| Xanthophylls (mg/kg) | . | . | 12.83 |
